# Toripalimab plus chemotherapy vs. chemotherapy in patients with advanced non-small-cell lung cancer: A cost-effectiveness analysis

**DOI:** 10.3389/fphar.2023.1131219

**Published:** 2023-02-14

**Authors:** Gengwei Huo, Wenjie Liu, Shuo Kang, Peng Chen

**Affiliations:** ^1^ Department of Thoracic Oncology, Tianjin Medical University Cancer Institute and Hospital, National Clinical Research Center for Cancer, Key Laboratory of Cancer Prevention and Therapy Of Tianjin, Tianjin’s Clinical Research Center for Cancer, Tianjin, China; ^2^ Department of Oncology, Jining No. 1 People’s Hospital, Jining, Shandong, China; ^3^ Medical Insurance Office, The Second Hospital of Hebei Medical University, Shijiazhuang, China

**Keywords:** toripalimab, cost-effectiveness, NSCLC, CHOICE-01, markov model

## Abstract

**Background:** The potency and safety of toripalimab combination with chemotherapy (TC) as the first-line therapy for advanced non-small cell lung cancer (NSCLC) have been demonstrated in the CHOICE-01 study. Our research explored whether TC was cost-effective compared to chemotherapy alone from the Chinese payer perspective.

**Materials and methods:** Clinical parameters were obtained from a randomized, multicenter, registrational, placebo-controlled, double-blind, phase III trial. Standard fee databases and previously published literature were used to determine costs and utilities. A Markov model with three mutually exclusive health statuses (progression-free survival (PFS), disease progression, and death) was used to predict the disease course. The costs and utilities were discounted at 5% per annum. The main endpoints of the model included cost, quality-adjusted life years (QALYs), and incremental cost-effectiveness ratio (ICER). Univariate and probabilistic sensitivity analyses were performed to investigate the uncertainty. Subgroup analyses were performed to verify the cost-effectiveness of TC in patients with squamous and non-squamous cancer.

**Results:** TC combination therapy yielded an incremental 0.54 QALYs with an incremental cost of $11,777, compared to chemotherapy, giving rise to ICERs of $21,811.76/QALY. Probabilistic sensitivity analysis revealed that TC was not favorable at 1 time GDP *per capita*. With a prespecified willingness-to-pay threshold (WTP) of three times the GDP *per capita*, combined treatment had a 100% probability of being cost-effective and had substantial cost-effectiveness in advanced NSCLC. Probabilistic sensitivity analyses showed that TC was more likely to be accepted with a WTP threshold higher than $22,195 in NSCLC. Univariate sensitivity analysis showed that the utility of PFS state, crossover proportions of the chemotherapy arm, cost per cycle of pemetrexed treatment, and discount rate were the dominant influencing factors. Subgroup analyses found that in patients with squamous NSCLC, the ICER was $14,966.09/QALY. In the non-squamous NSCLC, ICER raised to $23,836.27/QALY. ICERs were sensitive to the variance of the PFS state utility. TC was more likely to be accepted when WTP increases exceeded $14,908 in the squamous NSCLC subgroup and $23,409 in the non-squamous NSCLC subgroup.

**Conclusion:** From the perspective of the Chinese healthcare system, TC may be cost-effective in individuals with previously untreated advanced NSCLC at the prespecified WTP threshold compared to chemotherapy, and more significant in individuals with squamous NSCLC, which will provide evidence for clinicians to make the best decisions in general clinical practice.

## Introduction

Lung cancer developing from the bronchial mucosal epithelium and alveoli is still one of the most malignant neoplastic diseases, with the highest mortality and incidence (Bray et al., 2018; Miller et al., 2018). Non-small cell lung cancer (NSCLC) accounts for about 83% of lung cancer cases (Siegel et al., 2020). In fact, only 6% of patients with advanced NSCLC are alive 5 years after diagnosis (Topalian et al., 2019), the design of new treatment methods to improve survival is urgently needed. The treatment of lung cancer mainly includes surgical, radiotherapy and systemic drug therapy. The development of therapeutic drugs has experienced three eras, including the era of cytotoxic chemotherapy drugs, anti-angiogenic drugs, targeted drugs, and immunotherapy drugs emerging in recent years (Fisher and D'Orazio, 2000; Fukuoka et al., 2011; Brahmer et al., 2015; Garon et al., 2015; Sharma and Allison, 2015; Soria et al., 2018; Ramalingam et al., 2020). At present, the immune checkpoint inhibitors (ICIs) including programmed cell death protein 1 (PD-1) or its ligand 1 (PD-L1), and cytotoxic T-lymphocyte-associated protein 4 (CTLA-4) have been approved for certain types of cancer (Miller et al., 2019).

Toripalimab, a monoclonal antibody targeting PD-1 developed in China, was approved by the China Food and Drug Administration as the second-line therapy for unresectable or metastatic melanoma, locally advanced or metastatic urothelial cancer, and recurrent or metastatic nasopharyngeal cancer. In addition, it was approved as the first-line treatment for unresectable locally advanced or relapsed/metastatic esophageal squamous cell cancer, non-operable locally advanced or metastatic without epidermal growth factor receptor gene mutation (EGFR) and anaplastic lymphoma kinase gene fusions (ALK) non-squamous NSCLC, locally relapsed or metastatic nasopharyngeal cancer (Keam, 2019; Wang et al., 2020; Yang et al., 2020; Zhang et al., 2021). Some researches have indicated that chemotherapy could enhance the antitumor effect of the immune system, thereby enhancing immunotherapy activity and improving clinical efficacy (Bracci et al., 2014; Peng et al., 2015; Leonetti et al., 2019; Judd and Borghaei, 2020). Recently, the CHOICE-01 study evaluated the clinical benefit of toripalimab plus chemotherapy (TC) *versus* chemotherapy alone in advanced NSCLC (Wang et al., 2022). The findings indicated that the TC arm, compared to the chemotherapy arm, improved progression-free survival (PFS) [median 8.4 vs. 5.6 months; hazard ratio (HR), 0.49; 95%CI 0.39–0.61; *p* < 0.0001] and overall survival (OS) (median not reached (>24 months) vs. 17.1 months; HR, 0.69; 95%CI 0.53–0.92; *p* = 0.0099). The incidence of grade ≥3 treatment-related adverse events (AEs) was similar between the two arms (78.6% vs. 82.1%). Thus, adding toripalimab to chemotherapy appears to be a compelling first-line therapy for advanced NSCLC. Nevertheless, proper allocation of limited medical resources and consideration of cost-effectiveness in medical decision-making are needed by clinical decision-makers. The purpose of our research was to estimate the cost-effectiveness of TC *versus* chemotherapy alone in the first-line therapy of advanced NSCLC from the Chinese healthcare system perspective.

## Materials and methods

### Participants and interventions

We extracted basic clinical data from a randomized, multicenter, registrational, double-blind, placebo-controlled, phase III trial (CHOICE-01) (Wang et al., 2022). Eligible patients were untreated, without EGFR or ALK driver mutations, had locally advanced (stage IIIB or IIIC) or metastatic NSCLC, and were randomly divided (2:1) into the TC or chemotherapy arm. For non-squamous NSCLC, individuals received 4–6 cycles of pemetrexed 500 mg/m^2^ IV (intravenous injection) + carboplatin AUC 5 IV q3w plus toripalimab or placebo at a dose of 240 mg IV q3w, followed by maintenance of pemetrexed + toripalimab or placebo. For squamous NSCLC, individuals received 4–6 cycles of nab-paclitaxel 100 mg/m^2^ intravenously (IV) on days 1, 8, and 15 + carboplatin AUC 5 IV q3w plus toripalimab or placebo at a dose of 240 mg IV q3w, followed by toripalimab or placebo maintenance. As a result, 465 patients were randomly distributed to the TC or chemotherapy arm, stratified according to baseline demographics, with substantially balanced disease features between the two treatment arms (Wang et al., 2022). The baseline case analysis assumed that the maximum treatment time for toripalimab was 2 years. We assumed that all of the adenocarcinoma patients received first line 4 cycles of pemetrexed + carboplatin plus toripalimab or placebo, followed by maintenance of pemetrexed + toripalimab or placebo. All of the squamous cell carcinoma patients received 4 cycles of nab-paclitaxel + carboplatin plus toripalimab or placebo, followed by toripalimab or placebo maintenance. After disease progression, 51.1% of individuals in the TC arm and 83.3% of individuals in the chemotherapy alone arm received at least one subsequent treatment (Wang et al., 2022), while those in the chemotherapy alone arm were allowed to cross over to toripalimab monotherapy. Assuming that the individuals in the TC arm would no longer use other immunological drugs and switch to other chemotherapy regimens after disease progression, 4 cycles of docetaxel chemotherapy would be selected for subsequent treatment (Zhu et al., 2021). In the chemotherapy arm, we supposed that individuals who progressed would adopt docetaxel or toripalimab or best supportive care (BSC), which was consistent with the guidelines and the actual situation. Computed tomography was used to evaluate the tumor once every 6 weeks.

### Model framework

**FIGURE 1:**
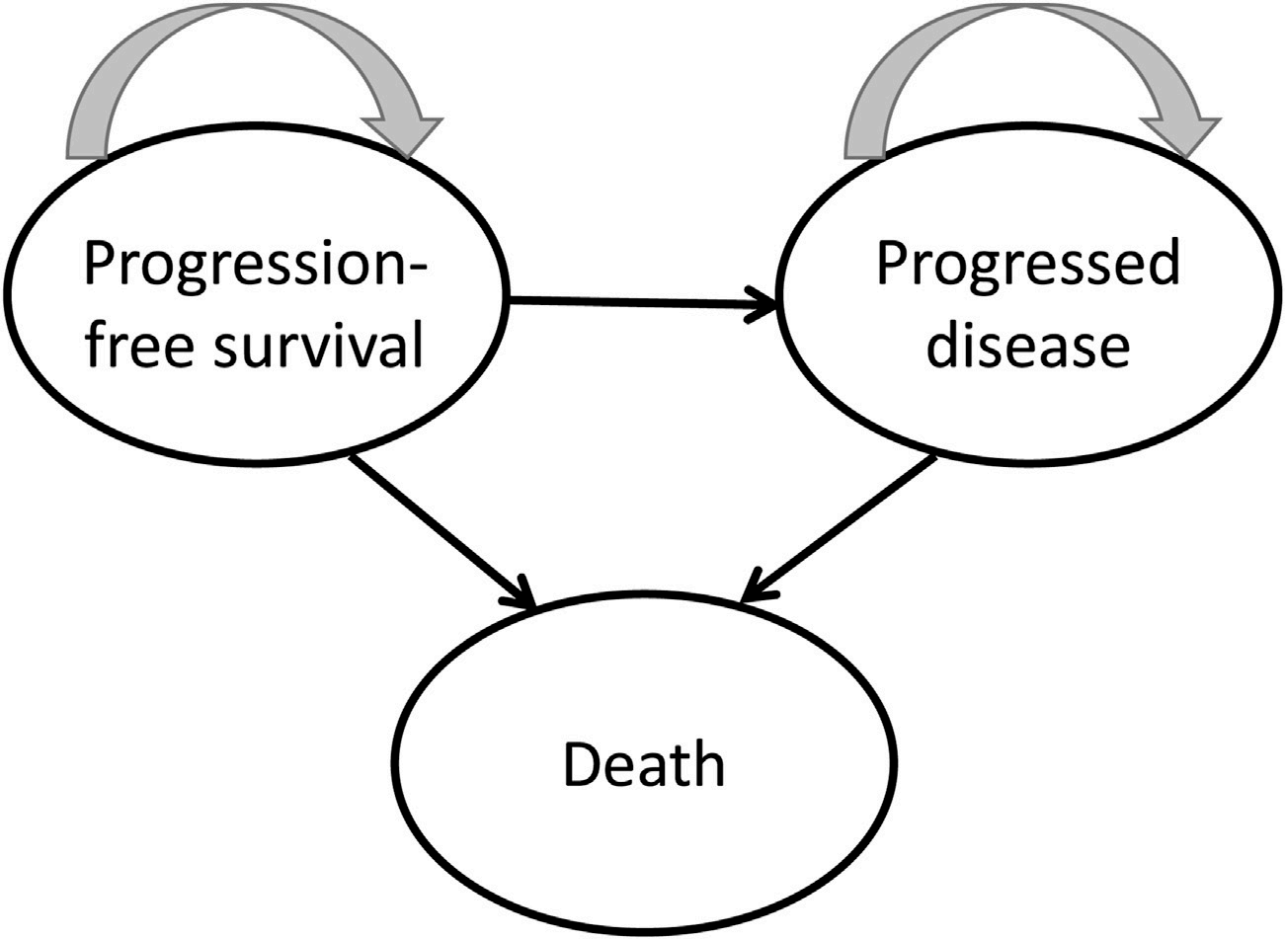
Partitioned survival model structure.

A mathematical Markov model was built using TreeAge Pro 2022 software to measure costs and utilities. Three mutually exclusive health states constituted the model structure: PFS, progressive disease (PD), and death (Figure 1). Almost all individuals in the two arms died after 10 years in the model simulation. Therefore, the time limit for our analysis was designed at 10 years (Cai et al., 2019; Liu et al., 2020; Weng et al., 2020). One cycle length in this model was defined as 21 days. Individuals were partitioned to each status according to the cumulative probabilities of PFS and OS and those stemming from the patient data from the CHOICE-01 study. All hypothetical individuals started out in a PFS status, receiving first-line therapy. If disease progression occurred, individuals entered PD status and received subsequent treatment until death.

### Clinical data

**FIGURE 2:**
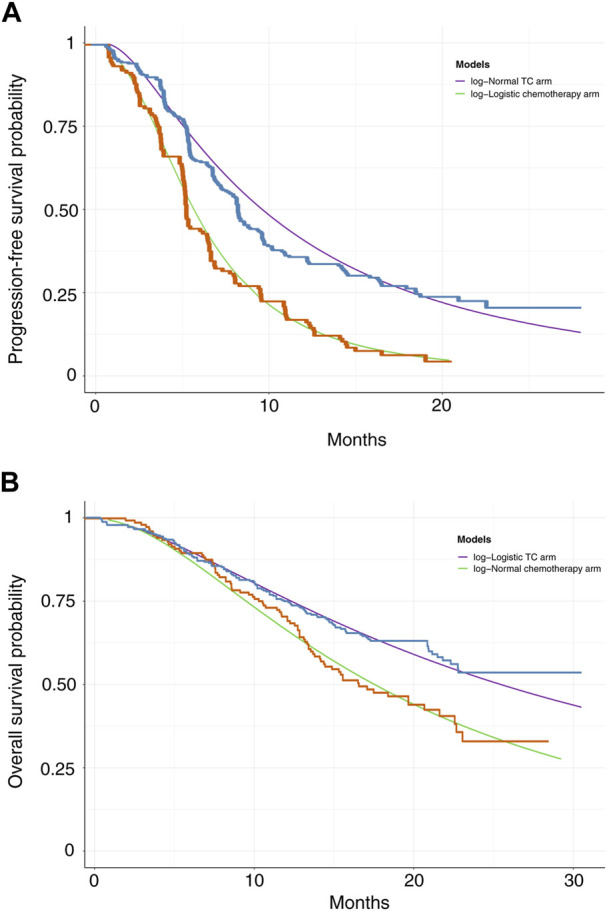
PFS **(A)** and OS **(B)** curves for the original trial and model estimated data in NSCLC.

The GetData Graph Digitizer software was utilized to extrapolate the transition probabilities over a lifetime horizon according to the PFS and OS curves for TC and chemotherapy alone from the CHOICE-01 trial (Hoyle and Henley, 2011). Survival functions such as exponential, Weibull, gamma, Gompertz, log-normal, and log-logistic distributions were fitted to the data from curves based on the Akaike and Bayesian information criterion (Kearns et al., 2019). Log-logistic distributions were selected for the PFS curve in the chemotherapy arm and OS curve in the TC arm, and log-normal distributions were selected for the PFS curve in the TC arm and OS curve in the chemotherapy alone arm (Supplementary Table S1). Based on different histological types, the distributions of parameters in the TC and chemotherapy arms were selected (Supplementary Tables S2, S3). The survival curve simulation is shown in Figure 2 and Supplementary Figures S1, S2. US life tables were used to assess the background mortality rate (Arias et al., 2017).

### Costs data

Health resource use and only direct medical expenditures were regarded, including those related to drug acquisition and administration, disease management, and treatment-related adverse events (AEs) (Table 1).

**TABLE 1:** Model parameters and distribution.

Variable	Baseline value (reference)	Range		Distribution
		Minimum	Maximum	
Log-normal PFS survival model with toripalimab + chemotherapy	Meanlog = 2.262; sdlog = 0.950	-	-	-
Log-logistic PFS survival model with placebo + chemotherapy	Shape = 2.489; scale = 6.089	-	-	-
Log-logistic OS survival model with toripalimab + chemotherapy	Shape = 1.510; scale = 25.348	-	-	-
Log-normal OS survival model with placebo + chemotherapy	Meanlog = 2.897; sdlog = 0.791	-	-	-
Subsequent chemotherapy proportions of toripalimab + chemotherapy arm	0.511 (Wang et al., 2022)	0.409	0.613	Beta
BSC in toripalimab + chemotherapy arm	0.489 estimated	0.3912	0.5868	Beta
Crossover proportions of chemotherapy arm	0.538 (Wang et al., 2022)	0.4304	0.6456	Beta
Subsequent chemotherapy proportions of chemotherapy arm	0.295 (Wang et al., 2022)	0.236	0.354	Beta
BSC in chemotherapy arm	0.167 estimated	0.1336	0.2004	Beta
Grade ≥3 AEs incidence in toripalimab + chemotherapy				
Anemia	0.299 (Wang et al., 2022)	0.2392	0.3588	Beta
Leukopenia	0.357 (Wang et al., 2022)	0.2856	0.4284	Beta
Neutropenia	0.555 (Wang et al., 2022)	0.444	0.666	Beta
Thrombocytopenia	0.172 (Wang et al., 2022)	0.1376	0.2064	Beta
Grade ≥3 AEs incidence in placebo + chemotherapy				
Anemia	0.359 (Wang et al., 2022)	0.2872	0.4308	Beta
Leukopenia	0.417 (Wang et al., 2022)	0.3336	0.5004	Beta
Neutropenia	0.538 (Wang et al., 2022)	0.4304	0.6456	Beta
Thrombocytopenia	0.179 (Wang et al., 2022)	0.1432	0.2148	Beta
Utility				
Progression-free disease	0.80 (Nafees et al., 2017)	0.64	0.96	Beta
Progressed disease	0.32 (Nafees et al., 2017)	0.26	0.38	Beta
AEs disutility				
Anemia	0.07 (Yang et al., 2021)	0.058	0.088	Beta
Leukopenia	0.20 (Yang et al., 2021)	0.112	0.168	Beta
Neutropenia	0.20 (Yang et al., 2021)	0.16	0.24	Beta
Thrombocytopenia	0.11 (Yang et al., 2021)	0.086	0.130	Beta
Drug cost, US$				
Toripalimab/cycle	375 (YAOZH.com, 2022)	300	375	Fixed in PSA
Carboplatin/cycle	55.18 (YAOZH.com, 2022)	44.15	66.22	Gamma
Nab-paclitaxel/cycle	122.73 (YAOZH.com, 2022)	98.182	147.273	Gamma
Pemetrexed/cycle	841.48 (YAOZH.com, 2022)	673.18	1009.78	Gamma
Docetaxel/cycle	31.60 (YAOZH.com, 2022)	25.28	37.93	Gamma
AEs cost, US$				
Anemia per event	571.98 (Yang et al., 2021)	457.58	686.38	Gamma
Leukopenia per event	451.11 (Yang et al., 2021)	360.89	541.33	Gamma
Neutropenia per event	496.46 (Yang et al., 2021)	397.17	595.75	Gamma
Thrombocytopenia per event	3820.77 (Yang et al., 2021)	3056.62	4584.92	Gamma
Administration cost, US$				
Cost of CT examination/1 time	56.05 (Shao et al., 2022)	44.84	67.27	Gamma
Cost of blood biochemical examination/1 time	45.34 (Shao et al., 2022)	36.27	54.41	Gamma
Cost of blood test/1 time	3.03 (Shao et al., 2022)	2.42	3.63	Gamma
Cost of urinalysis/1 time	0.61 (Shao et al., 2022)	0.49	0.73	Gamma
Physician Fee/1 day	3.03 (Shao et al., 2022)	2.42	3.63	Gamma
Cost of intravenous injection/1 day	1.67 (Shao et al., 2022)	1.33	2.00	Gamma
Cost of care/1 day	3.63 (Shao et al., 2022)	2.91	4.36	Gamma
Cost of bed/1 day	6.36 (Shao et al., 2022)	5.09	7.63	Gamma
Cost of terminal care per patient	2241.18 (Rui et al., 2022)	1792.94	2689.41	Gamma
BSC/cycle	122.18 (Li et al., 2020)	97.74	146.62	Gamma
Follow-up visit	77.01 (Shao et al., 2022)	61.61	92.41	Gamma
Patients’ body surface area, m^2^	1.72 (Yang et al., 2021)	1.38	2.06	Normal
Discount rate (%)	5 (Yang et al., 2021)	0	8	Fixed in PSA

BSC, best supportive care; PFS, progression-free survival; OS, overall survival; AEs, adverse effects; CT, computed tomography.

Acquisition costs for toripalimab, carboplatin, nab-paclitaxel, pemetrexed, and subsequent treatments were obtained from public databases, which were all the latest in 2022 (Shao et al., 2022; YAOZH.com, 2022). The cost of drug management was equal to the cost of the chemotherapy drug preparation injection plus the cost of hospitalization. According to the published literature, the one-time cost of end-of-life care per patient who died was $2,241.18 (Rui et al., 2022), best supportivecare cost per cycle was $122.18 (Li et al., 2020). We only regarded severe AEs (grade ≥3) with an incidence of greater than 5%, involving anemia, neutropenia, leukopenia, and thrombocytopenia (Wang et al., 2022). The AEs costs were extracted from published articles (Yang et al., 2021). For each therapeutic regimen, the total expenditure per AE was calculated based on the incidence of AE and its related unit cost. It is assumed that after the occurrence of AEs, patients are treated only in the first cycle, and the cost of AE occurs only once. Drug dosage was calculated according to a body surface area of 1.72 m^2^ and creatinine clearance of 70 mL/min (Goulart and Ramsey, 2011; Wu et al., 2011). Suppose that the corresponding expense is incurred at the beginning of each cycle; thus, there is no cost adjustment for the half cycle (Chen et al., 2022). From January to September 2022, the exchange rate of Chinese Yuan renminbi was 6.6 yuan per US dollar average. Total costs and quality-adjusted life-years (QALYs) were the primary outcomes, and a 5% discount rate per year was adopted in our analysis (Yang et al., 2021).

### Health-state utilities

The QALYs for different therapies were assessed. The health utility scores of PFS, PD, and death status were extracted from two health status utility studies on Chinese individuals with NSCLC, with 0.80, 0.32, and 0, respectively (Nafees et al., 2008; Nafees et al., 2017). AEs resulting in disutility values were also calculated in our analysis (Tolley et al., 2013; Nafees et al., 2017; Wan et al., 2019). The decline in the overall QALY related to all AEs was applied to the first cycle of the models (Su et al., 2021). All the parameters associated with the utilities are displayed in Table 1.

### Univariate sensitivity analysis and probabilistic sensitivity analysis

Sensitivity analyses were performed to examine the impact of the parameter uncertainty on the outcomes. The imported data and ranges of the sensitivity analyses are displayed in Table 1. Clinical parameters in univariate sensitivity analyses were varied over plausible ranges based on ±20% for body surface area, body weight, costs and health state utilities, with discount rate ranging from 0% to 8%, as shown in the tornado diagram. In light of real-world performance, there is no possibility that the price of toripalimab will rise; therefore, only the impact of the price slide on the incremental cost-effectiveness ratio (ICER) was conducted. Probabilistic sensitivity analysis (PSA) applied a Monte Carlo simulation of 1,000 individuals to evaluate the best strategy under various hypothetical willingness-to-pay (WTP) thresholds through simultaneous and random preset parameter variations. Scatter plots and cost-effectiveness acceptability curves (CEACs) were applied to analyze the cost-effectiveness of each option with different WTP threshold (Rabin and de Charro, 2001; Lin et al., 2020). In 2021, the Chinese *per capita* GDP was $12,269 (NBSC, 2022), so prespecified WTP was $36,807, which was three times the *per capita* GDP according to the WHO. PFS and OS parameters were obtained from the corresponding parametric survival distributions. AE disutilities and costs were derived from gamma distributions, and proportion, utility and probability from beta distributions.

### Subgroup analysis

PFS and OS curve of patients with adenocarcinoma and squamous cell carcinoma were extracted from the CHOICE-01 study respectively. Therapeutic regimen and the proportion of subsequent regimens in each subgroup was the same as the baseline case analysis respectively.

## Results

The median PFS and interim OS values obtained in our simulation were consistent with those in the CHOICE-01 study (Supplementary Table S4). Our model assessed median PFS of 8.4 months in the TC arm and 5.6 months in the chemotherapy arm, respectively. Based on data derived from the CHOICE-01 study, the median PFS was 8.4 months in the TC arm and 5.6 months in the chemotherapy arm. Our models assessed the interim OS analysis of not reached (>24 months) and 17.2 months for the TC and chemotherapy arms, respectively. It compared with OS of not reached (>24 months) and 17.1 months in the TC and chemotherapy arms, respectively based on the CHOICE-01 study. For different histological types, the median PFS values and interim OS analysis values for the TC and the chemotherapy arm are shown in Supplementary Tables S5, S6.

### Baseline analyses

Within a 10-year time horizon based on the Markov model, the total costs were $27,971 and $16,194 for the TC and placebo plus chemotherapy arms, respectively. The TC therapy yielded 1.44 QALYs and the chemotherapy yielded 0.90 QALYs. Therefore, individuals in the TC arm spent an additional $11,777 and produced an increase of 0.54 QALYs, giving rise to an ICER of $ 21,812 per QALY, which was higher than the one-time GDP *per capita*, but it was within the prespecified WTP threshold ($36,807/QALY), suggesting that TC therapy was economical compared to chemotherapy alone (Table 2).

**TABLE 2:** Base-case results of the model.

Patients	Arm	Costs, US$	△Costs, US$	QALYs	△QALYs	ICER US$/QALY
Overall	Placebo + Chemotherapy	16,194	-	0.90	-	-
	Toripalimab + Chemotherapy	27,971	11,777	1.44	0.54	21,811.76
Squamous	Placebo + Chemotherapy	11,278	-	0.82	-	-
	Toripalimab + Chemotherapy	16,817	5,539	1.19	0.37	14,966.09
non-squamous	Placebo + Chemotherapy	20,513	-	0.90	-	-
	Toripalimab + Chemotherapy	42,397	21,884	1.82	0.92	23,836.27

ICER: incremental cost-effectiveness ratio; QALY: quality-adjusted life-years.

## Sensitivity analysis

### Univariate sensitivity analyses

**FIGURE 3:**
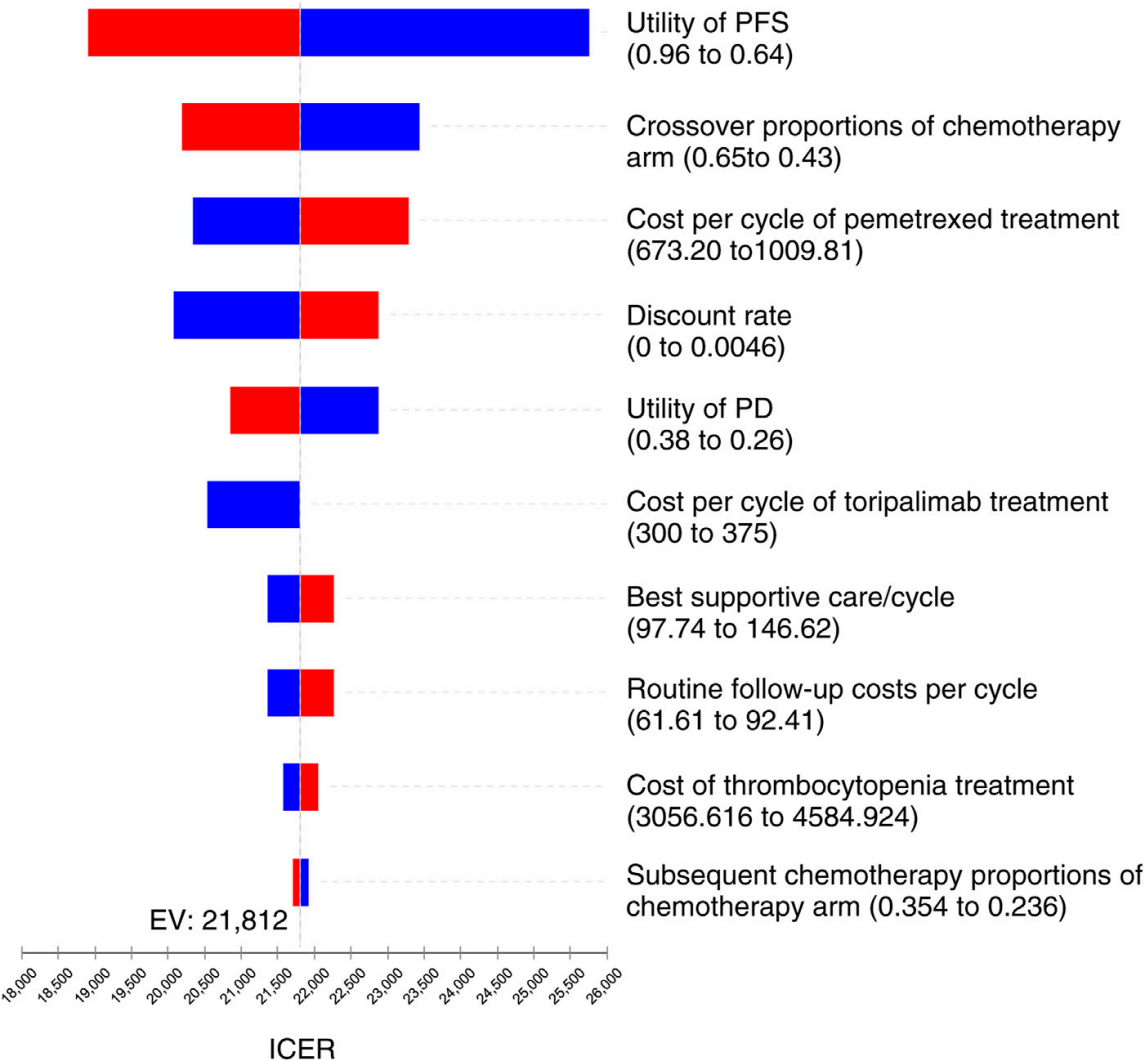
Tornado diagram for univariate sensitivity analyses in NSCLC. It summarized the results of one-way sensitivity analysis, which listed influential parameters in descending order according to their effect on the ICER over the variation of each parameter value.

As the tornado diagram for patients with NSCLC in Figure 3 displays, the utility of PFS status, crossover proportions of the chemotherapy arm, cost per cycle of pemetrexed treatment, and discount rate were the dominant influencing factors in this research. Nevertheless, there is no intersection between the generated ICER and WTP when all parameters vary within the corresponding ranges, indicating that the model is generally robust.

### Probabilistic sensitivity analysis

**FIGURE 4:**
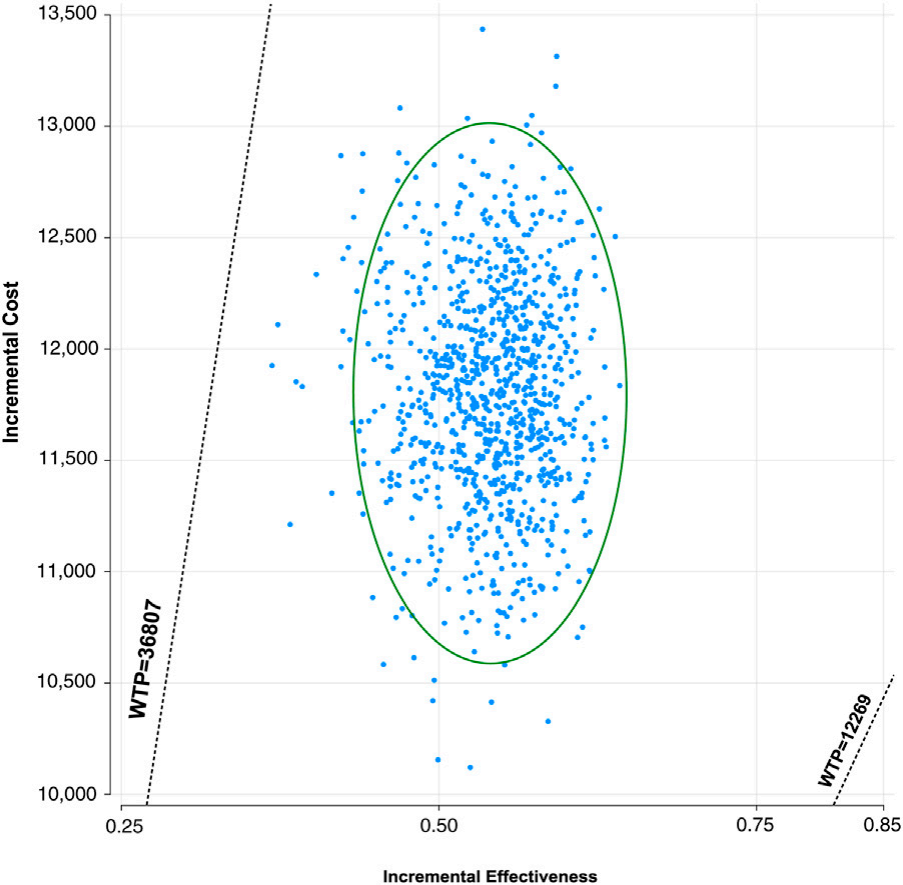
Incremental cost-effectiveness scatter plot diagram for toripalimab in combination with chemotherapy vs. chemotherapy alone in NSCLC. Each dot represents the ICER for 1 simulation. An ellipse means 95% confidence interval.

**FIGURE 5:**
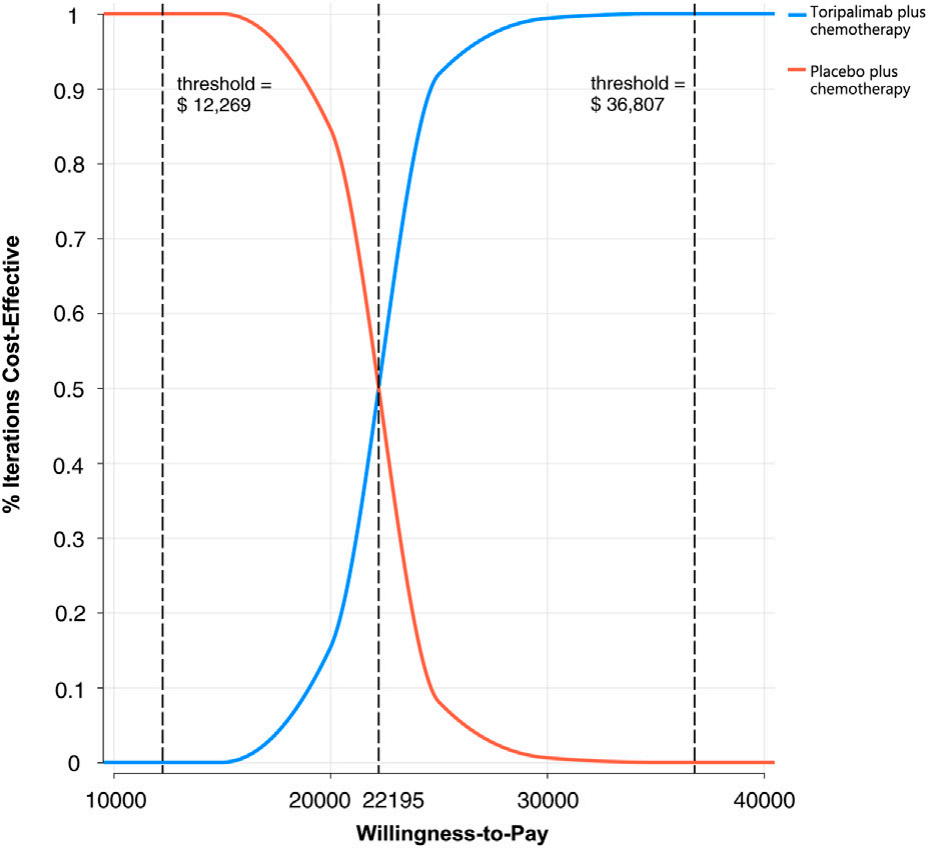
The cost-effectiveness acceptability curves for probabilistic sensitivity analyses in NSCLC.

A Monte Carlo simulation of 1,000 patients showed that the scatter points were located in the first quadrant of the coordinate axis, indicating that TC may produce more QALYs but at a higher cost. When WTP was set at one-time GDP *per capita*, all of the scatter points of ICER are located above the WTP line. When WTP was set at three times the GDP *per capita*, all scatter points were located below the WTP line (Figure 4). As shown in Figure 5, the CEACs indicated that TC had a 100% probability of being cost-effective when the designated WTP threshold was $36,807 per QALY compared to placebo plus chemotherapy. TC was unfavorable when the WTP thresholds is below $22,195.

### Subgroup analysis

Among the subgroups of individuals with squamous NSCLC, the cumulative costs and effectiveness were $16,817 and 1.19 QALYs in TC arm, and $11,278 and 0.82 QALYs in the placebo plus chemotherapy arm, respectively, and the ICER was $14,966.09/QALY (Table 2). ICERs were most sensitive to the variations of the utility of PFS status, crossover proportions of chemotherapy arm, discount rate and cost of toripalimab per cycle (Supplementary Figure S3). Among the subgroup of individuals with non-squamous NSCLC, the cumulative costs and effectiveness were $42,397 and 1.82 QALYs in the TC arm, and $20,513 and 0.90 QALYs in the placebo plus chemotherapy arm, and the ICER was $23,836.27/QALY (Table 2). ICERs were the most sensitive to variations in the utility of PFS status, cost of pemetrexed per cycle, utility of PD status, and discount rate (Supplementary Figure S4). PSA revealed that TC was more likely to be accepted with a WTP threshold higher than $14,908 in squamous NSCLC subgroup and higher than $23,409 in the non-squamous NSCLC subgroup. TC had a cost-effectiveness probability of 16% and 0% in squamous and non-squamous NSCLC, respectively, when the WTP threshold was set at one-time GDP *per capita*. With a WTP of three times the GDP *per capita*, TC therapy had substantial cost-effectiveness (Supplementary Figures S5–S8). A subgroup analysis based on histological type revealed that TC was more cost-effective in individuals with squamous NSCLC.

## Discussion

To our knowledge, this study is the first to synthesize the latest evidence to estimate the economic results of toripalimab in NSCLC using an economic modeling method. Currently, drug development with favorable curative potency and few adverse effects is the principal focus of research and development. The report on the clinical benefits of limited course immunotherapy plus chemotherapy in CHOICE-01 trial was of great interest to the oncologists and patients (Wang et al., 2022). Nevertheless, the pricing of antineoplastic drugs must be both effective and affordable. We assessed the cost-effectiveness of TC in advanced NSCLC as a first-line therapy due to the increasing interest and enormous unmet demand in the economic evaluation of new drugs (Uyl-de Groot and Löwenberg, 2018).

Based on our base-case analysis results, our analysis indicated that TC cost more ($27,971 *versus* $16,194) and produced more health outcomes than placebo plus chemotherapy (1.44 *versus* 0.90 QALYs), giving rise to ICERs of $21,811.76/QALY. Thus, TC was not favorable with a WTP threshold of $12,269 per QALY *versus* chemotherapy alone. While WTP threshold increased to $36,807 per QALY, TC had a probability of 100% to be cost-effectiveness. It spelled that the combination therapy may be a possibly effective and cost-effective choice for NSCLC individual with a higher WTP. Univariate sensitivity analysis and PSA both suggested that these results were robust. We also found that TC was equally cost-effective in individuals with different histological types, due to favorable ICERs ($14,966.09/QALY for squamous NSCLC; $23,836.27/QALY for non-squamous NSCLC) in our subgroup analysis.

The utility of PFS state, crossover proportions of the chemotherapy arm, cost per cycle of pemetrexed treatment, and discount rate were the dominant influencing factors in our analysis. With the extensive changes of these parameters, the TC therapy still has substantial cost-effectiveness when the WTP threshold is three times of GDP *per capita*. In the subsequent therapy after disease progression, we hypothesized that individuals in the TC arm were cross-treated with chemotherapy, and those in the chemotherapy arm were cross-treated with toripalimab. According to the CHOICE-01 trial, crossover proportions of the chemotherapy arm had a considerable influence because this parameter could affect the total cost of disease progression. In addition, the high cost of pemetrexed per cycle also has a substantial impact on the sensitivity analysis, which might be associated with the longer duration of the progression-free status in terms of the OS of individuals, as we know, pemetrexed are needed to apply for both first-line and maintenance therapy in individuals with non-squamous NSCLC, thus decreasing the price of pemetrexed may be an effective strategy to reduce ICER.

Several cost-effectiveness studies on the combination of immunotherapy and chemotherapy as the first-line therapy of NSCLC have been carried out (Zeng et al., 2019; Ding et al., 2020; Lin et al., 2020; Wu and Lu, 2020). An economic evaluation from China based on the CameL-sq trial showed similar results that camrelizumab, another anti-PD-1 humanized monoclonal drug, combined with chemotherapy in previously untreated squamous NSCLC, produced additional 0.47 QALYs and the accompanying incremental costs of $6,347.81 giving rise to an ICER of $13,571.68/QALY *versus* chemotherapy alone, and was significantly cost-effective at a WTP threshold of $38,184 per QALY (Shao et al., 2022). Although the combination of immunotherapy and chemotherapy as first-line in the CameL-sq was different from the CHOICE-01 trial, both of the two PD-1 inhibitors were indicated similar clinical benefits and pricing. The conclusion was consistent and comparable with our results ($14,966.09/QALY). Adding immunotherapy on the basis of limited course of chemotherapy could quickly control the condition of illness and avoid serious chemical toxicity at the same time, which has become a new treatment choice for advanced NSCLC. Reasonable economic assessment has been an indispensable part of the allocation of cancer treatment resources, and useful and helpful in the clinical management of the disease.

Toripalimab might open up opportunities for individuals with advance NSCLC to realize OS benefit. The price of toripalimab is lower than that of imported immunotherapy drugs because of the lower transportation costs, therefore, which is more readily available and widely used in Chinese patients. Our analysis provides evidence of cost-effectiveness that could have important policy and practical significance for reducing the medical burden, providing new ideas on how to increase the affordability of great-value innovative medicines. However, economic development in China’s provinces is uneven, and the WTP of a region needs to be considered when evaluating the cost-effectiveness of TC therapy. TC was favorable when 1 time GDP *per capita* was set as the WTP in Macao and Hongkong Special Administrative Region, Taiwan district, Beijing, and Shanghai. But not favorable when 3 times GDP *per capita* was set as the WTP in Heilongjiang and Gansu province. In addition, each country has different healthcare systems, costs, and modeling methods, and the conclusions summarized from one country may not be suited to another (Goldstein et al., 2015). Second, the results of our analysis were robust, as the sensitivity analysis displayed. The conclusions were more accurate than the standard survival model because of the flexible parametric modelings used to fit and extrapolate the survival data. It might be useful for patients, physicians, and policymakers to make treatment decisions based on the economic information from our subgroup analysis. Therefore, our cost-effectiveness finding gives a valuable and compelling reference for the selection of first-line therapy options for NSCLC.

This analysis has some limitations. First, it is inevitable to extrapolate the survival curve to acquire complete survival results owing to the short follow-up time of the CHOICE-01 study. The results of the actual survival curves could not be fitted entirely by the reconstructed survival curves. Nevertheless, the objective of adjusting the transition probability is to approach the real results as closely as possible. Second, the results concerning TC might have been exaggerated because grade 1 or 2 AEs were not considered and if the same AE occurs multiple times for the same patient, assumed that patient is counted only once when calculating the number of adverse events in our analysis. From our univariate sensitivity analyses, the disutilities and costs related to AEs were minor; nevertheless, these AEs could not be neglected in our general clinical practice. Third, generalizability might be affected because the costs and WTP thresholds varied between different countries and medical centers. The results were still robust as varying parameters within the range of ±20% by sensitivity analysis. Moreover, the research simulated findings were originated from a randomized clinical trial but not from prospective real world study. The more mature the available data, the more stable the model. Future work needs to be conducted to illustrate whether our model-based and trial-based outcomes can be simulated with long follow-up in real-world settings.

In summary, our analysis estimated the cost-effectiveness of TC compared with chemotherapy alone in previously untreated individuals with advanced NSCLC and indicated that TC is a cost-effective choice for a Chinese-payer perspective. Furthermore, subgroup analysis based on histological type showed that TC was more cost-effective in individuals with squamous NSCLC, which could be regarded in the decision-making process to propose treatment suggestions for individuals with advanced NSCLC. However, due to some limitations of this article, further long term follow-up outcomes and real-world data are demanded.

## Data Availability

The original contributions presented in the study are included in the article/Supplementary Material, further inquiries can be directed to the corresponding author.
