# Supplementary material for: Toripalimab plus chemotherapy vs. chemotherapy in patients with advanced non-small-cell lung cancer: A cost-effectiveness analysis
Source: Front Pharmacol. 2023 Feb 14;14:1131219. doi: 10.3389/fphar.2023.1131219 (PMC9971805; doi:10.3389/fphar.2023.1131219)
Supplement: Supplementary file 1 [file Table1.DOC]

Table S1. AIC and BIC for different parameter distributions of PFS and OS in NSCLC.

| Distribution | PFS | | | | OS | | | |
| --- | --- | --- | --- | --- | --- | --- | --- | --- |
|  | Toripalimab + chemotherapy arm | | Placebo + chemotherapy arm | | Toripalimab + chemotherapy arm | | Placebo + chemotherapy arm | |
|  | AIC | BIC | AIC | BIC | AIC | BIC | AIC | BIC |
| Exponential | 1433.455 | 1437.188 | 800.3404 | 803.3903 | 1125.955 | 1129.688 | 721.6075 | 724.6574 |
| Gamma | 1408.783 | 1416.249 | 755.0397 | 761.1394 | 1116.963 | 1124.430 | 691.9374 | 698.0371 |
| Gompertz | 1434.53 | 1441.997 | 786.4302 | 792.5299 | 1124.827 | 1132.294 | 707.2401 | 713.3398 |
| Weibull | 1417.428 | 1424.895 | 763.9637 | 770.0634 | 1118.334 | 1125.800 | 695.2257 | 701.3254 |
| Log-logistic | 1388.399 | 1395.866 | 747.7537 | 753.8534 | 1114.906 | 1122.372 | 690.7100 | 696.8097 |
| Log-normal | 1388.158 | 1395.625 | 747.9314 | 754.0311 | 1115.250 | 1122.717 | 688.7100 | 694.8097 |

Table S2. AIC and BIC for different parameter distributions of PFS and OS in squamous NSCLC.

| Distribution | PFS | | | | OS | | | |
| --- | --- | --- | --- | --- | --- | --- | --- | --- |
|  | Toripalimab + chemotherapy arm | | Placebo + chemotherapy arm | | Toripalimab + chemotherapy arm | | Placebo + chemotherapy arm | |
|  | AIC | BIC | AIC | BIC | AIC | BIC | AIC | BIC |
| Exponential | 691.0334 | 694.0239 | 375.9244 | 378.2148 | 646.5408 | 649.5313 | 329.9293 | 332.2197 |
| Gamma | 676.3861 | 682.3670 | 326.9526 | 331.5335 | 639.7538 | 645.7347 | 305.8437 | 310.4246 |
| Gompertz | 692.3542 | 698.3351 | 352.0127 | 356.5936 | 644.6431 | 650.6240 | 316.1452 | 320.7261 |
| Weibull | 681.7816 | 687.7625 | 333.7639 | 338.3449 | 640.4961 | 646.4769 | 307.9367 | 312.5176 |
| Log-logistic | 663.8323 | 669.8132 | 322.3353 | 326.9162 | 638.7980 | 644.7789 | 303.9415 | 308.5225 |
| Log-normal | 666.6045 | 672.5854 | 326.0153 | 330.5962 | 641.3763 | 647.3572 | 306.5171 | 311.0980 |

Table S3. AIC and BIC for different parameter distributions of PFS and OS in nonsquamous NSCLC.

| Distribution | PFS | | | | OS | | | |
| --- | --- | --- | --- | --- | --- | --- | --- | --- |
|  | Toripalimab + chemotherapy arm | | Placebo + chemotherapy arm | | Toripalimab + chemotherapy arm | | Placebo + chemotherapy arm | |
|  | AIC | BIC | AIC | BIC | AIC | BIC | AIC | BIC |
| Exponential | 754.8229 | 757.9105 | 434.6119 | 437.0308 | 498.3708 | 501.4584 | 399.5734 | 401.9922 |
| Gamma | 747.2992 | 753.4743 | 423.9008 | 428.7385 | 497.7804 | 503.9556 | 392.5459 | 397.3835 |
| Gompertz | 756.7481 | 762.9233 | 431.1723 | 436.0100 | 500.1421 | 506.3173 | 398.7210 | 403.5587 |
| Weibull | 750.6347 | 756.8099 | 426.0125 | 430.8502 | 498.2455 | 504.4207 | 394.1988 | 399.0365 |
| Log-logistic | 738.1609 | 744.3361 | 424.2498 | 429.0875 | 496.8275 | 503.0027 | 391.3930 | 396.2307 |
| Log-normal | 736.7234 | 742.8986 | 421.002 | 425.8397 | 495.6929 | 501.8681 | 388.4448 | 393.2824 |

Table S4. The median progression-free survival and interim overall survival analysis observed in the CHOICE-01 trial and estimated by the current cost-effectiveness model in NSCLC.

| Treatment | Model | Trial of CHOICE-01 | Difference |
| --- | --- | --- | --- |
| Median progression-free survival, mo |  |  |  |
| Toripalimab + chemotherapy arm | 8.4 | 8.4 | 0 |
| Placebo + chemotherapy arm | 5.6 | 5.6 | 0 |
| Median overall survival, mo |  |  |  |
| Toripalimab + chemotherapy arm | >30 | >30 | - |
| Placebo + chemotherapy arm | 17.2 | 17.1 | 0.1 |

Table S5. The median progression-free survival and interim overall survival analysis observed in the CHOICE-01 trial and estimated by the current cost-effectiveness model in squamous NSCLC.

| Treatment | Model | Trial of CHOICE-01 | Difference |
| --- | --- | --- | --- |
| Median progression-free survival, mo |  |  |  |
| Toripalimab + chemotherapy arm | 8.2 | 8.1 | 0.1 |
| Placebo + chemotherapy arm | 5.6 | 5.6 | 0 |
| Median overall survival, mo |  |  |  |
| Toripalimab + chemotherapy arm | 21.0 | 21.0 | 0 |
| Placebo + chemotherapy arm | 17.2 | 17.6 | 0.4 |

Table S6. The median progression-free survival and interim overall survival analysis observed in the CHOICE-01 trial and estimated by the current cost-effectiveness model in nonsquamous NSCLC.

| Treatment | Model | Trial of CHOICE-01 | Difference |
| --- | --- | --- | --- |
| Median progression-free survival, mo |  |  |  |
| Toripalimab + chemotherapy arm | 9.6 | 9.7 | 0.1 |
| Placebo + chemotherapy arm | 5.5 | 5.5 | 0 |
| Median overall survival, mo |  |  |  |
| Toripalimab + chemotherapy arm | >30 | >30 | - |
| Placebo + chemotherapy arm | 17.0 | 17.0 | 0 |
