# Supplementary material for: Toripalimab plus chemotherapy vs. chemotherapy in patients with advanced non-small-cell lung cancer: A cost-effectiveness analysis
Source: Front Pharmacol. 2023 Feb 14;14:1131219. doi: 10.3389/fphar.2023.1131219 (PMC9971805; doi:10.3389/fphar.2023.1131219)
Supplement: Supplementary file 2 [file DataSheet1.PDF]

A

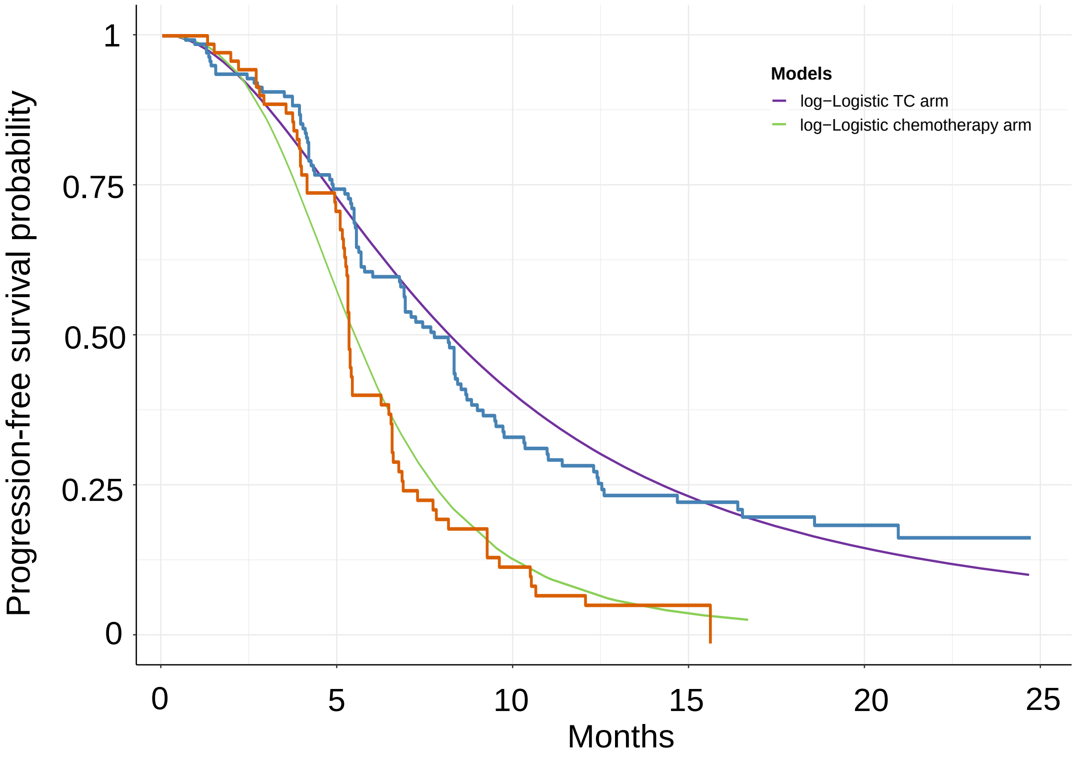

B

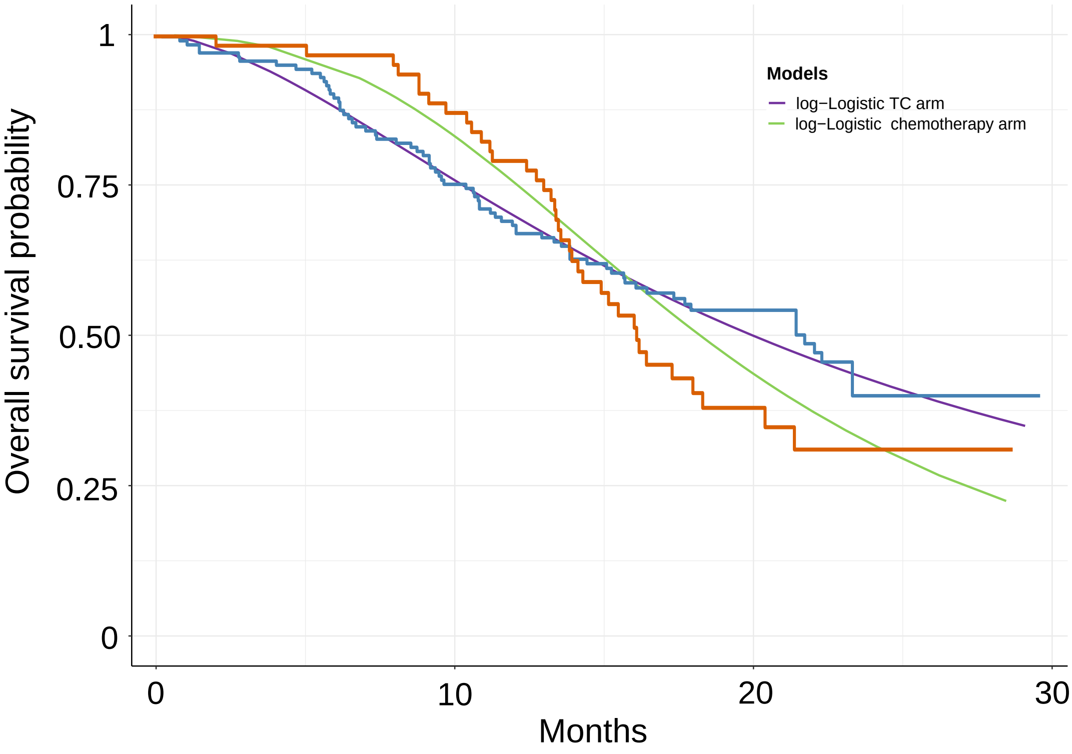

Figure S1 PFS (A) and OS (B) curves for the original trial and model estimated data in squamousNSCLC.

A

B

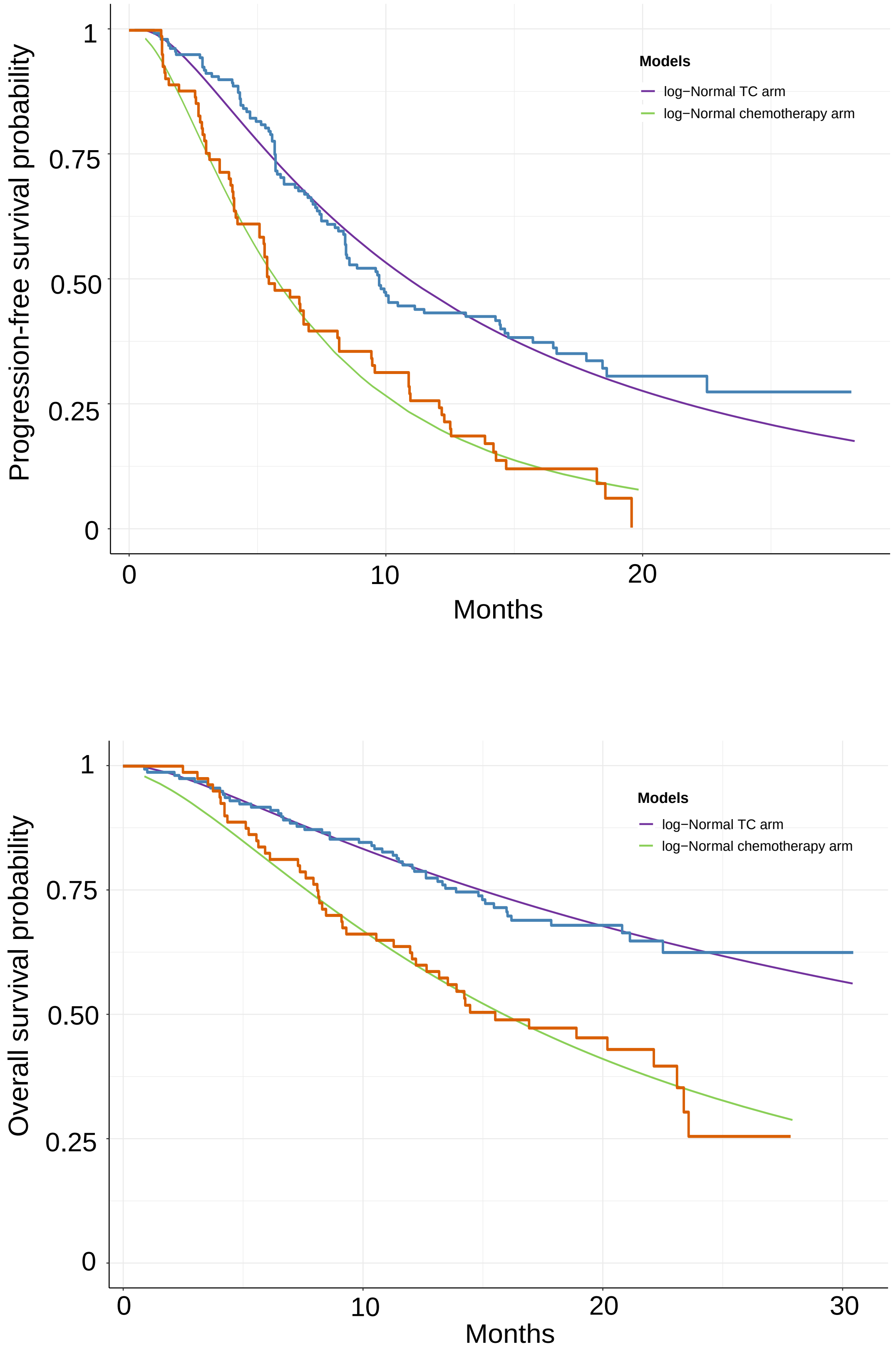

Figure S2 PFS (A) and OS (B) curves for the original trial and model estimated data in nonsquamousNSCLC.

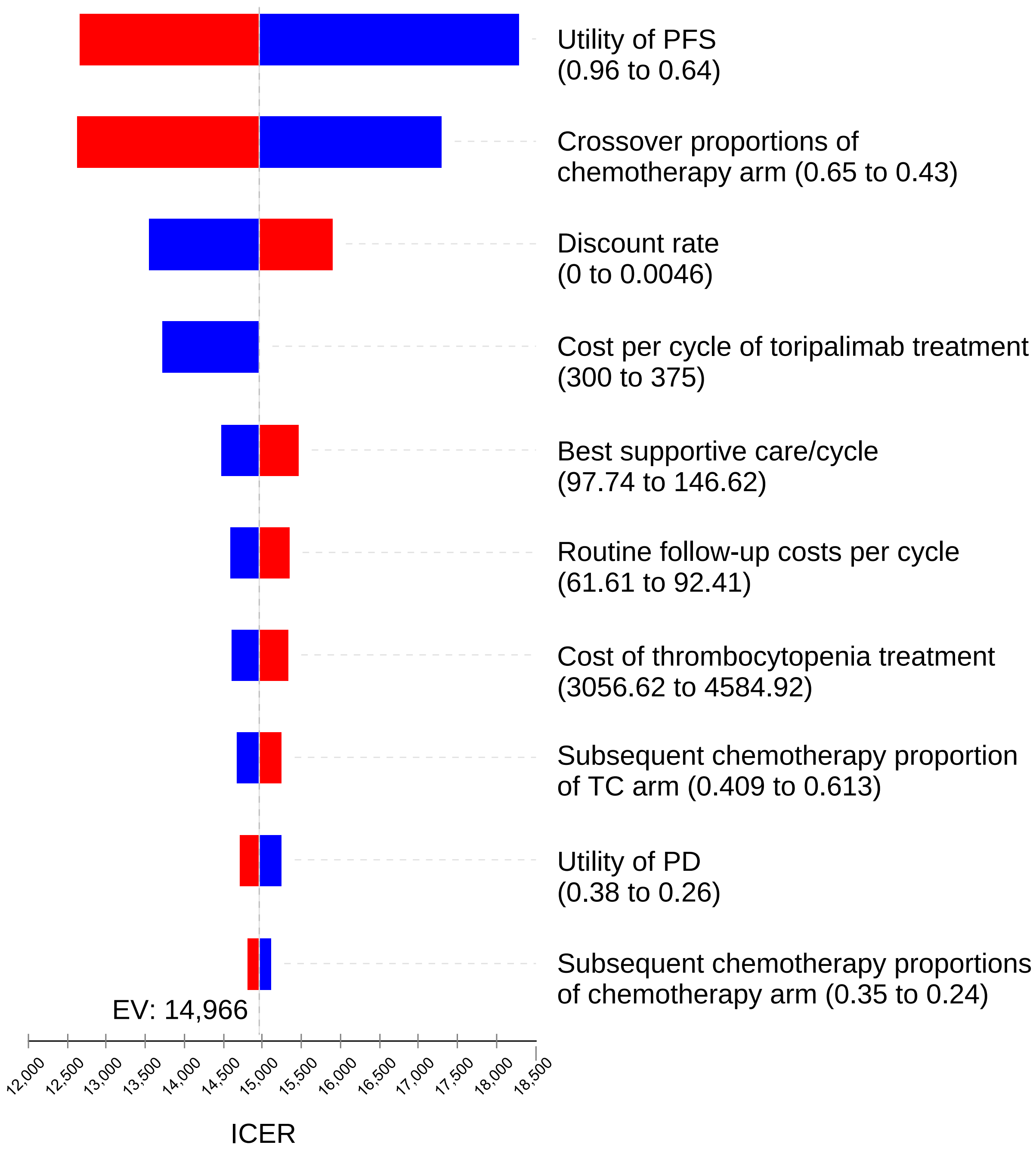

Figure S3 Tornado diagram for univariate sensitivity analyses in squamous NSCLC. It summarized the results of one-way sensitivity analysis, which listed influential parameters in descending order according to their effect on the ICER over the variation of each parameter value.

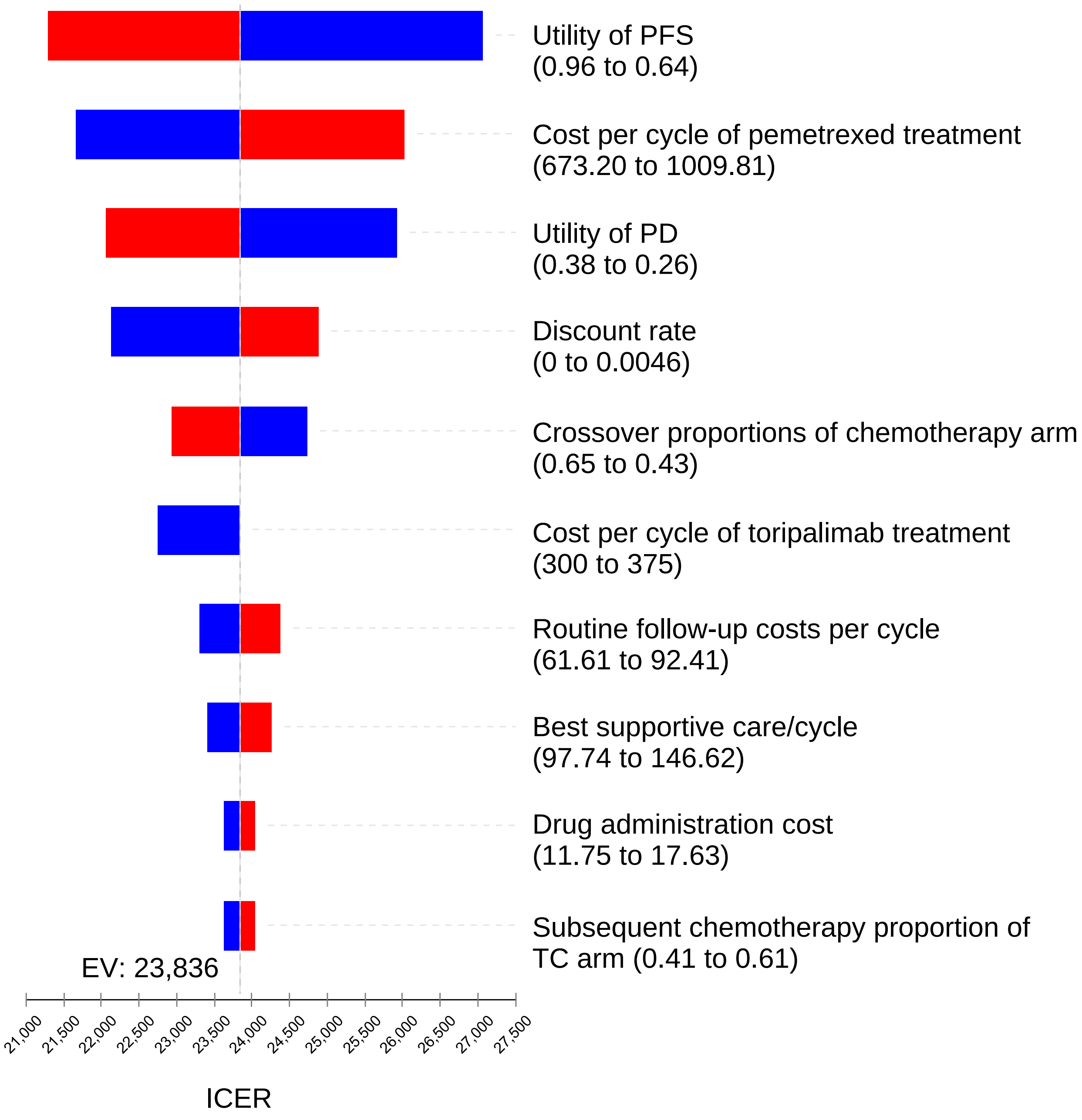

Figure S4 Tornado diagram for univariate sensitivity analyses in nonsquamous NSCLC. It summarized the results of one-way sensitivity analysis, which listed influential parameters in descending order according to their effect on the ICER over the variation of each parameter value.

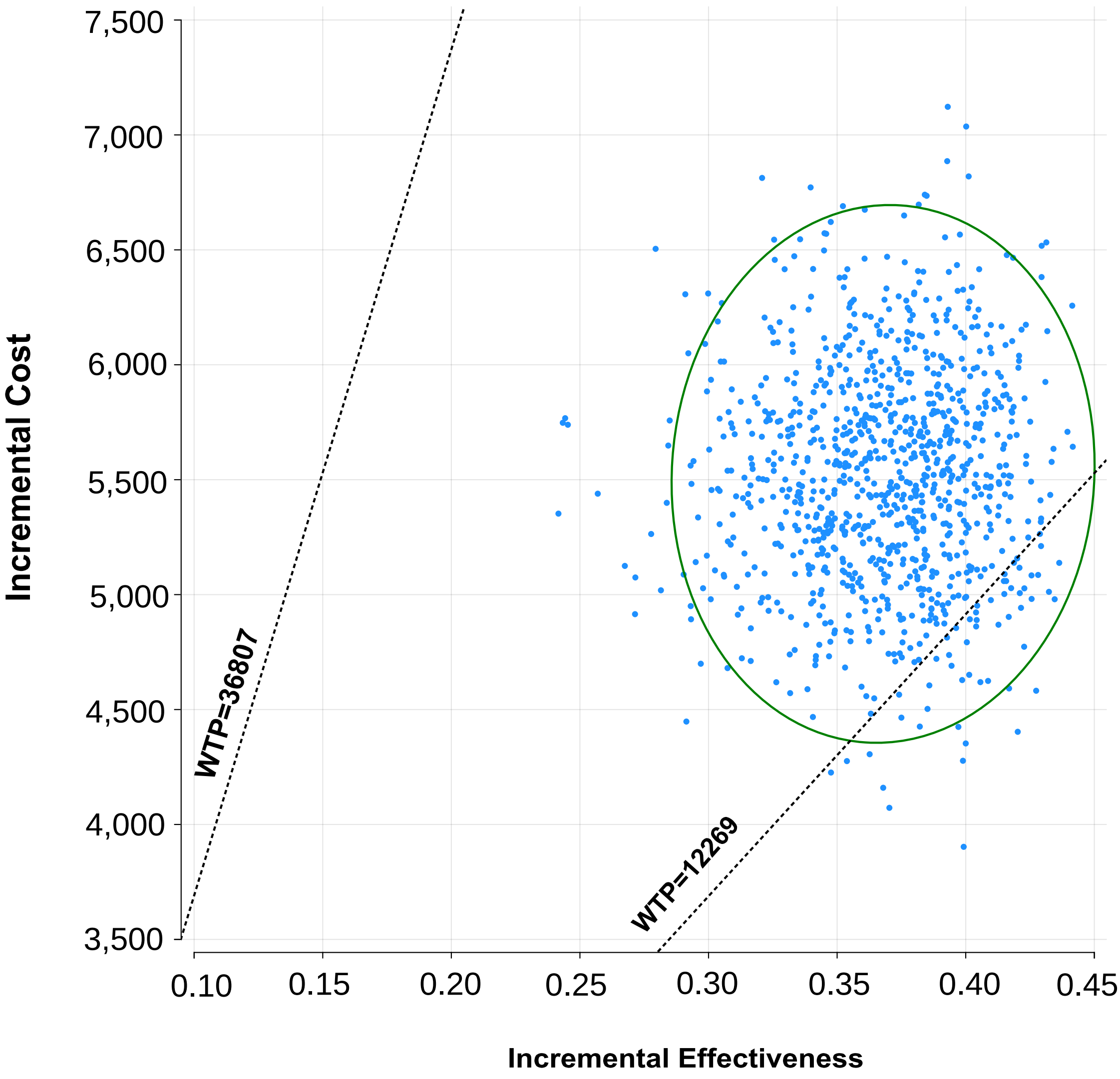

Figure S5 Cost-effectiveness scatter plot in squamous NSCLC. Each dot represents the ICER for 1 simulation. An ellipse means 95% confidence interval.

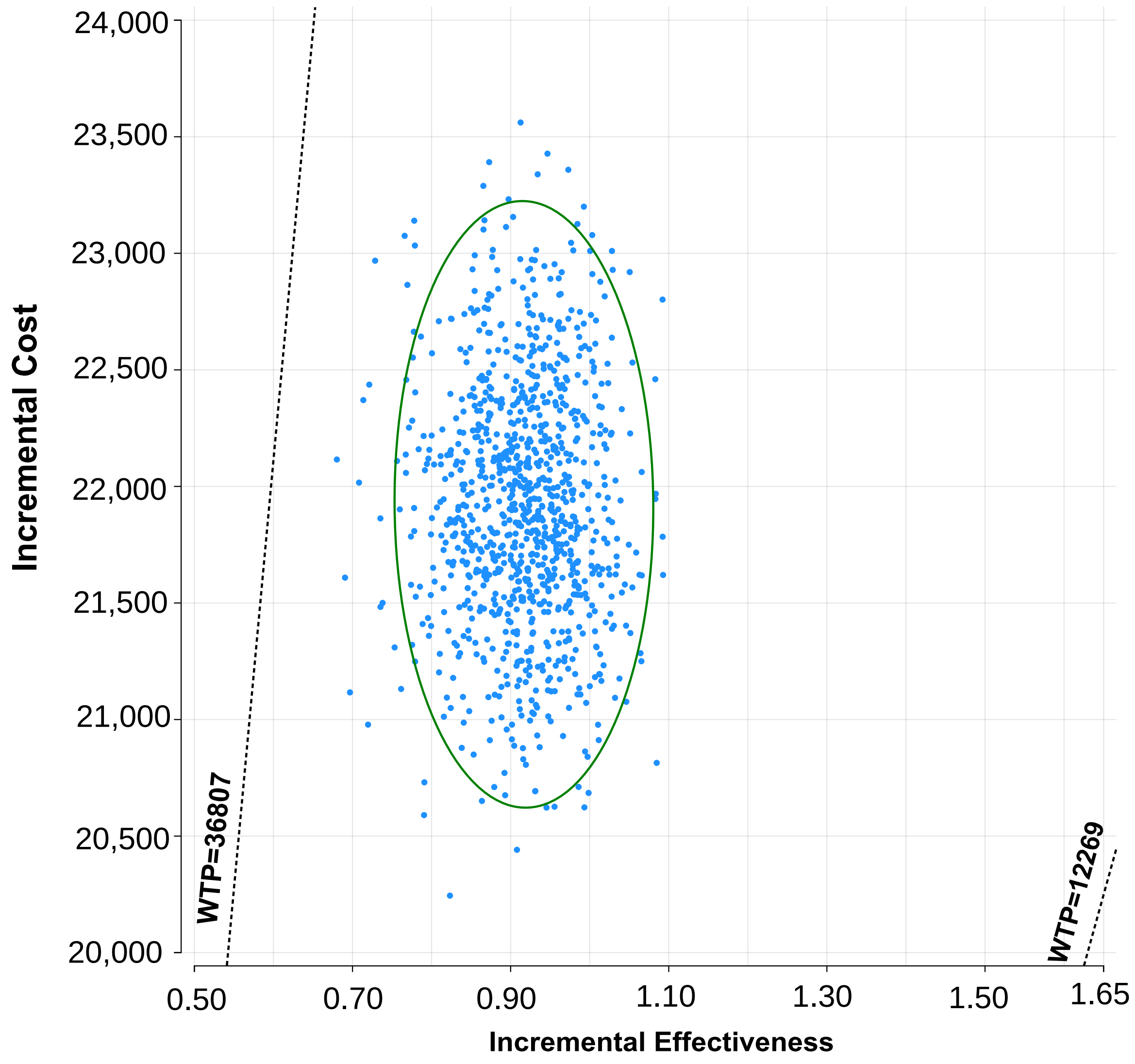

Figure S6 Cost-effectiveness scatter plot in nonsquamous NSCLC. Each dot represents the ICER for 1 simulation. An ellipse means 95% confidence interval.

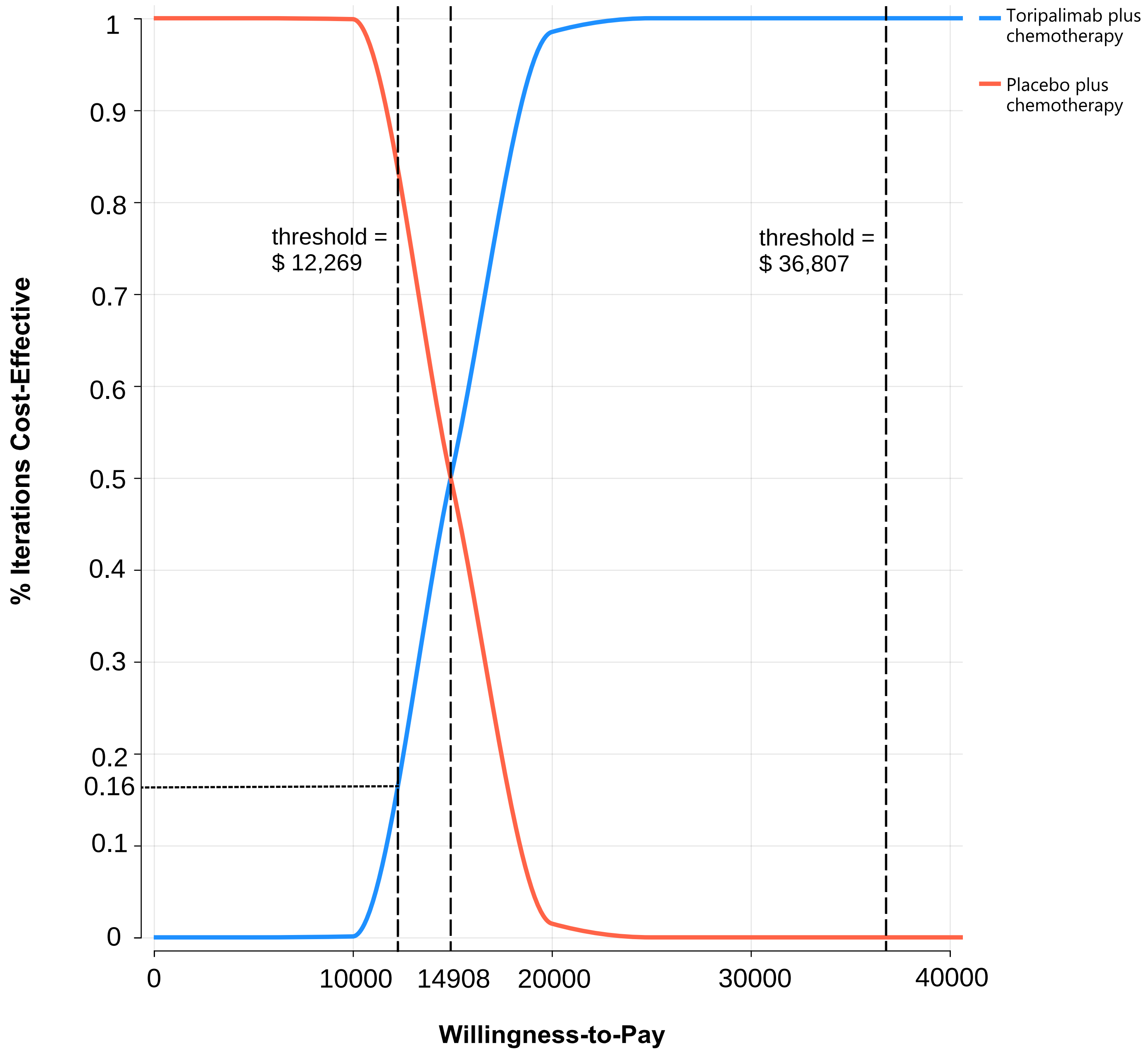

Figure S7 Cost-effectiveness acceptability curve in squamous NSCLC.

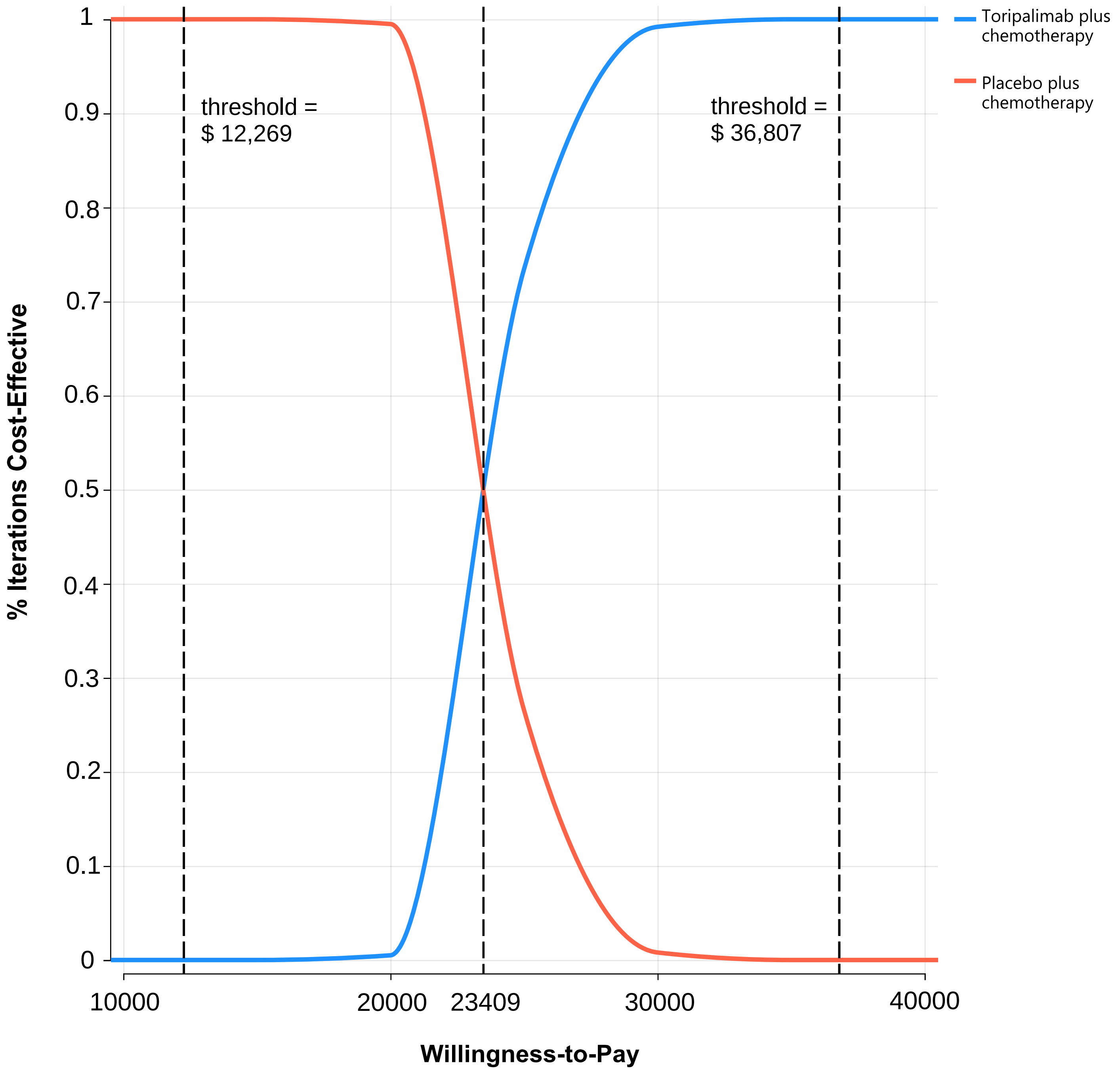

Figure S8 Cost-effectiveness acceptability curve in nonsquamous NSCLC.
